# Re-infection rates and clinical outcomes following arthrodesis with intramedullary nail and external fixator for infected knee prosthesis: a systematic review and meta-analysis.

**DOI:** 10.1186/s12891-018-2283-4

**Published:** 2018-10-10

**Authors:** Giovanni Balato, Maria Rizzo, Tiziana Ascione, Francesco Smeraglia, Massimo Mariconda

**Affiliations:** 1 Department of Public Health, Section of Orthopaedic Surgery, “Federico II” University, Via S. Pansini 5, Building 12, 80131 Naples, Italy; 2 Department of Infectious Diseases, D. Cotugno Hospital - AORN dei Colli, Naples, Italy

**Keywords:** Periprosthetic joint infection, Intramedullary nail, External fixator, Knee arthrodesis, Knee arthroplasty, Re-infection

## Abstract

**Background:**

Knee arthrodesis with intramedullary (IM) nail or external fixator (EF) is the most reliable therapeutic option to achieve definitive infection control in patients with septic failure of total knee arthroplasty (TKA). The first aim of this study was to compare re-infection rates following knee arthrodesis for periprosthetic joint infection (PJI) with IM nail or EF. The second aim was to compare rates of radiographic union, complication, and re-operation as well as clinical outcomes.

**Methods:**

A systematic search was performed in electronic databases for longitudinal studies of PJIs (minimum ten patients; minimum follow-up = 1 year) treated by knee arthrodesis with IM nail or EF. Studies were also required to report the rate of re-infection as an outcome measure. Eligible studies were meta-analyzed using random-effect models.

**Results:**

The rate (95% confidence intervals) of re-infection was 10.6% (95% CI 7.3 to 14.0) in IM nail arthrodesis studies. The corresponding re-infection rate for EF was 5.4% (95% CI 1.7 to 9.1). This difference was significant (*p* = 0.009). The use of IM nail resulted in more advantages than EF for frequency of major complications and limb shortening. Other postoperative clinical and radiographic outcomes were similar for both surgical strategies.

**Conclusions:**

The available evidence from the aggregate published data suggests that knee arthrodesis with EF in the specific context of PJI has a reduced risk of re-infection in comparison with the IM nail strategy. The use of IM nail is more effective for the complication rate and shortening of the affected limb.

## Background

Periprosthetic joint infection (PJI) is one of the most serious complications of total knee arthroplasty (TKA). Two-stage revision is considered as the most effective surgical technique for treating chronic PJI of the knee [1], but the one-stage revision has been recently gaining popularity [2]. These revision strategies have a re-infection rate of 8.8% and 7.6%, respectively [1]. Risk factors for the development of recurrent infection after revision surgery include isolation of difficult micro-organisms [3, 4], comorbidities [3], and previous surgeries [4]. Once any measures to salvage a functional TKA through multiple revision procedures have been exhausted, knee arthrodesis or the above-knee amputation represent the only options to eradicate the infection. Amputation should only be performed in conditions of severe and irreversible damage of the bone and soft-tissues [2, 5] because of its unsatisfactory functional results [6, 7]. Conversely, knee arthrodesis can provide acceptable quality of life and functionality of the knee when there is sufficient residual bone stock [8]. Currently, the external fixator (EF) and intramedullary (IM) nail represent preferred methods to achieve knee arthrodesis in the context of septic failure of TKA [9]. Published results of these two surgical strategies have been variable [9–13]. Ideally, to compare EF and IM nail for knee arthrodesis would require evidence from carefully designed randomized clinical trials that are unlikely to occur given the low PJI event rates recorded after TKA [1]. The lack of robust evidence from randomized clinical trials results in uncertainty on the effectiveness of these surgical options. Hence, there is a need for further work to compare these strategies for arthrodesis. Using a meta-analytic approach, our first aim was precisely to evaluate the effectiveness of IM nail and EF knee arthrodesis adopting re-infection rate as the primary endpoint. Other relevant outcomes including the rate of radiographic union, complication, and re-operation as well as the postoperative limb length discrepancy (LLD), pain, and functional status were also investigated. Moreover, we aimed to compare and describe the differences in these outcomes between the two surgical options.

## Methods

### Data sources and search strategy

We searched for studies investigating different outcomes following knee arthrodesis performed with IM nail and/or EF in MEDLINE, Scopus, EMBASE, Web of Science, and Cochrane databases from inception up to September 2017. The Preferred Reporting Items for Systematic Review and Meta-Analyses (PRISMA) [14] methodology guidance was employed. The search strategy used a combination of the following key words: Knee arthroplasty OR Knee replacement OR Knee prosthesis AND Infection OR Septic AND Nail OR Fixator AND Arthrodesis OR Fusion. No language restrictions were employed. The reference lists of selected articles were also examined for any additional articles not identified from the database search.

### Eligibility criteria

We included longitudinal studies comprising of consecutive unselected patients affected by PJI who were treated by knee arthrodesis using an IM nail or EF. We excluded: (i) studies that reported on these surgical methods in selected group of patients (such as patients with a specific infection or single preoperative diagnosis); (ii) studies with less than 1 year of minimum follow-up; (iii) studies with less than 10 participants; and (iiii) studies including patients with knee arthrodesis for causes of TKA failure different from infection where the outcome in septic patients could not be specifically assessed.

### Study assessment and data extraction

Initial screening of titles and abstracts was performed by two pairs of independent reviewers (GB and MR, FS and TA). Full text was obtained for all abstracts that appeared to meet the inclusion criteria or where there was any uncertainty. Each article was assessed by two independent reviewers (GB, FS) using the inclusion criteria and any discrepancies regarding the eligibility of an article were solved with a third author (MM). Thereafter, relevant data were extracted from each included study. Two authors (MR, TA) performed quality assessment of eligible articles using the Methodological Index for Non-Randomized Studies (MINORS) criteria [15]. MINORS is a valid instrument designed to assess the methodological quality of non-randomized surgical studies. It yields a maximum score of 16 and 24, respectively, for non-comparative and comparative studies.

### Statistical analysis

The rate of re-infection (i.e. number of re-infections at follow-up/total number of participants) with 95% confidence interval (CI) represented the primary outcome. Secondary outcomes were the rate of radiographic union, surgical complication, and re-operation as well as several clinical findings recorded at follow-up including the quality of life (SF-36 or SF-12 Questionnaire), functionality of knee (Oxford Knee score, Knee Society score etc.), severity of pain (Visual analog scale), and LLD. Subgroup analysis was undertaken, based on the effect of different types of IM nail and EF on different outcomes (re-infection rate, fusion rate, and time to fusion). Two broad types of IM nail (i.e. long and short) were identified. For EF arthrodesis, the subgroup analysis compared the effect of unilateral vs biplanar/circular and pins vs wires EF. Heterogeneity between studies was tested using the I^2^ statistic (0% to 40% = not relevant; 30% to 60% = moderate; 50% to 90% = substantial; 75% to 100% = considerable) [14]. The primary and secondary outcomes were pooled using random effects models to account for the effect of between-study heterogeneity. Due to the unsuitability of pooling data for LLD, knee functional scales, the pain severity, and quality of life questionnaires, these outcomes were assessed using a comparison of means. A two-sample t test and chi-square test were used to test the significance of cross-sectional differences between the IM nail and EF knee arthrodesis and between different subgroups of surgical implant. We utilized Open Meta Analyst (Center for Evidence Synthesis, RI, USA) and SPSS version 23 (SPSS, Chicago, IL, USA) for all statistical analyses. *P* ≤ 0.05 was considered significant.

## Results

**Fig. 1:**
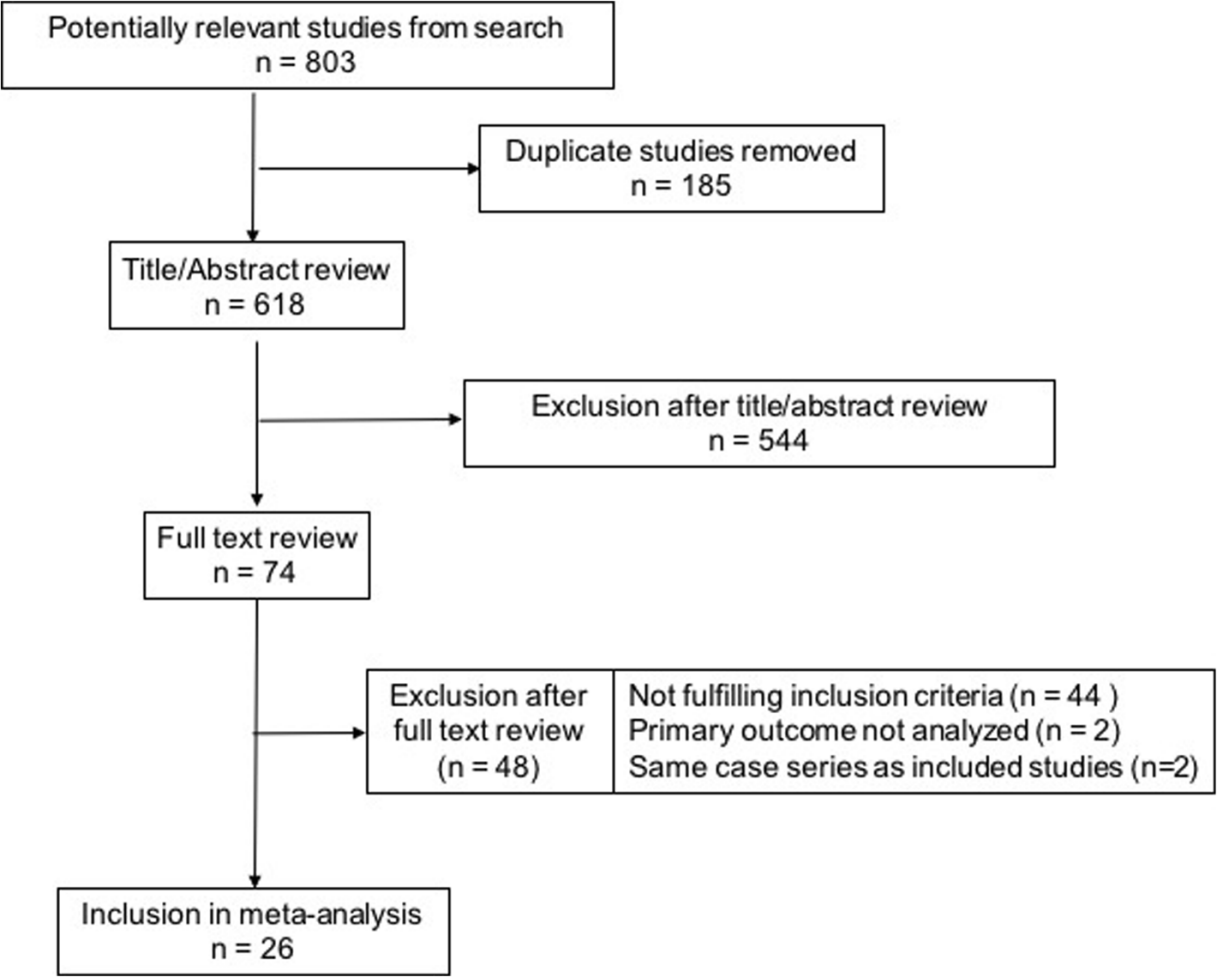
Literature search and methodology of selection

The flow diagram of our search strategy is reported in Fig. 1. The computer search and manual screening of reference lists of relevant studies identified 803 potentially relevant citations. After initial screening based of titles and abstracts, 74 articles remained for full text evaluation. After detailed assessment, we excluded 48 references. The remaining 26 articles [5, 10, 12, 13, 16–37] were included in the meta-analysis. Two of these [10, 22] were retrospective studies comparing outcomes of nail and EF arthrodesis. Hence, data from 18 and 10 studies were used for the assessment of surgical results of knee arthrodesis with IM nail and EF, respectively (Table 1).

**Table 1:** Characteristics of the studies included in the systematic review and meta-analysis

Lead Author, Publication Date	Location	Year of study	Mean age (years)	% male	Mean follow up (months)	Participants *n*	Two-stage/ one-stage	Surgical implant	Re-infections/ persisting infections n (%)	Fusion rate % (time to fusion - months)	Major complications *n*	Reoperations *n*	Postoperative limb length discrepancy (mm)	Quality score
Intramedullary nail														
Hungerer et al., 2017 [26]	Germany	2003–2012	68.6	43	53 (12–119)	55	NS	Short modular nail	12 (21.8%)	NS	13	20	18	9/16
Friedrich et al., 2017 [23]	Germany	2008–2014	70.2	NS	31 (12–74)	37	37/0	Short modular nail	5 (13.5%)	NS	2	7	22	10/16
Hawi et al., 2015 [5]	Germany	2002–2012	68.8	63	67 (24–143)	27	0/27	Short modular cemented nail	4 (14.8%)	NS	0	3	NS	11/16
Röhner et al., 2015 [12]	Germany	1997–2013	68	31	71 (12–204)	26	26/0	Short modular cemented nail	13 (50%)	NS	0	13	NS	10/16
Miralles-Muñoz et al., 2014 [29]	Spain	2001–2010	74.6	14	50 (36–60)	29	29/0	Short modular cemented nail	2 (6.9%)	NS	4	3	8	12/16
Scarponi et al., 2014 [33]	Italy	2000–2011	65	47	62 (24–105)	38	38/0	Short modular cementless nail	4 (10.5%)	NS	5	5	13	9/16
Iacono et al., 2013 [10]	Italy	2004–2009	69.3	NS	34(13–72)	21	21/0	Short modular cementless nail	3 (14.3%)	NS	0	3	8	9/16
Putman et al., 2013 [31]	France	2005–2008	67	NS	50 (28–90)	31	25/6	Short modular cementless nail	6 (19.4%)	68 (NS)	10	3	10	10/16
Barton et al., 2008 [17]	UK	1993–2004	NS	NS	53	10	9/1	Short modular nail	1 (10%)	100 (8)	2	2	NS	8/16
Talmo et al., 2007 [34]	USA	NS	67	41	48 (13–114)	29	25/4	Long modular cementless nail	4 (13.8%)	83 (6)	2	5	30	11/16
Bargiotas et al., 2006 [16]	USA	1999–2003	68	42	49 (18–72)	12	12/0	Long locked cementless nail	2 (16.6%)	83 (5.5)	2	2	55	10/16
Crockarell and Mihalko, 2005 [19]	USA	1991–2000	70.5	40	68 (24–96)	10	9/1	Long locked cementless nail	1 (10%)	100 (NS)	NS	NS	37	9/16
Domingo et al., 2004 [22]	Spain	1990–2001	67.8	50	Minimum 1 year	10	10/0	Short locked cementless nail	1 (10%)	90 (5)	2	1	NS	8/16
Volpi et al., 2004 [35]	France	1997–2001	68.6	43	19	12	12/0	Short modular cementless nail	2 (16.6%)	100 (3.6)	1	1	NS	7/16
Gore and Gassner, 2003 [24]	USA	1977–2001	71.5	56	54 (12–288)	16	14/2	Long Kuntscher or modular nail	2 (12.5%))	87.5 (NS)	3	4	NS	8/16
Waldman et al., 1999 [36]	USA	NS	64	38	29 (24–90)	21	21/0	Short modular cementless nail	0 (0%)	95 (6.3)	2	2	NS	8/16
Lai et al., 1998 [28]	Taiwan	1988–1994	68.2	43	47 (18–94)	28	8/20	Short locked cementless nail	1 (3.6%)	93 (5.2)	2	1	25	9/16
Wilde and Stearns, 1989 [37]	USA	1983–1988	65	33	Minimum 24	10	10/0	Long Kuntscher nail	3 (30%)	60 (6.6)	6	6	36	8/16
External fixator														
Balci, 2016 [13]	Turkey	1999–2012	67	18	51	17	17/0	Unilateral EF	1 (6%)	94 (7.6)	4	6	29	11/16
Corona et al., 2013 [18]	Spain	2004–2009	81	48	50 (12–81)	21	0/21	Unilateral EF	3 (14%)	81 (10.3)	NS	NS	47	9/16
Iacono et al., 2013 [10]	Italy	2001–2004	68.5	NS	93(82–110)	10	10/0	Semi circular Hoffmann EF	0 (0%)	90 (6.7)	1	2	45	9/16
Reddy et al., 2011 [32]	India	2003–2010	62.2	63	46 (12–84)	16	3/16	Ilizarov frame	1 (6%)	94 (6.6)	6	6	44	9/16
Parratten et al., 2007 [30]	France	1990–2003	64.8	43	88 (12–162)	14	6/8	2 unilateral EF	0 (0%)	93 (7.3)	2	2	45	9/16
Klinger et al., 2006 [27]	Germany	1990–2002	66.2	61	56 (24–132)	18	11/7	Unilateral EF	1 (6%)	83 (6.3)	3	2	36	9/16
Domingo et al., 2004 [22]	Spain	1990–2001	69.1	45	Minimum 1 year	11	11/0	Unilateral EF (3)	0 (0%)	100 (6.7)	1	0	NS	8/16
								Biplanar EF (8)		100 (6.1)				
David et al., 2001 [20]	Israel	1988-	65.9	39	41 (18–60)	10	NS	Ilizarov frame	0 (0%)	100 (6.4)	1	0	37	8/16
Hak et al., 1995 [25]	USA	1973–1990	58.5	45	43 (12–186)	20	NS	Unilateral EF (7)	1 (5%)	57 (7.1)	13	4	NS	8/16
								Biplanar EF (13)		62 (7.1)				
De Cloedt et al., 1994 [21]	Belgium	1984–1992	67.7	40	68 (14–108)	15	NS	2 unilateral EF	8 (53%)	67 (7.3)	NS	8	NS	8/16

*NS* Not stated, *EF* External fixator

### Re-infection

**Fig. 2:**
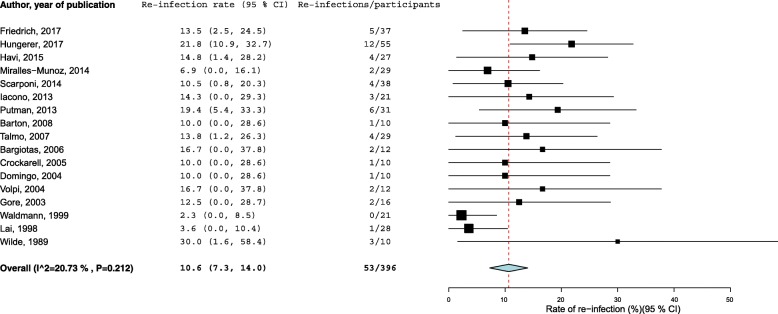
Rates of re-infection in patients treated by knee arthrodesis with intramedullary nail (1 outlier removed [12]). The summary estimates presented were calculated using random-effects models; CI, confidence interval (bars)

**Fig. 3:**
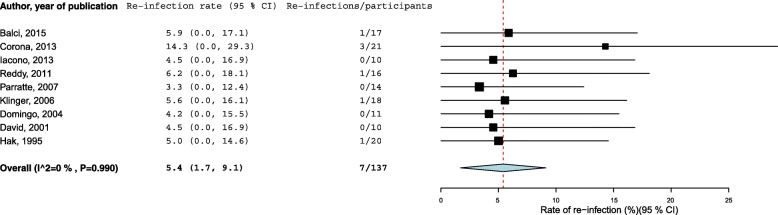
Rates of re-infection in patients treated by knee arthrodesis with external fixator (1 outlier removed [21]). The summary estimates presented were calculated using random-effects models; CI, confidence interval (bars)

Studies reporting on re-infection outcome after IM nail arthrodesis included 422 participants with 66 re-infections at follow-up. The pooled random-effects re-infection rate was 13.3% (95% CI 8.7 to 17.8, *p* < 0.001). There was moderate heterogeneity between the contributing studies (I^2^ = 54%; *p* = 0.004). On the exclusion of one single outlier study [12], the pooled re-infection rate decreased to 10.6% (95% CI 7.3 to 14.0, p < 0.001) and the heterogeneity was not significant (Fig. 2). There was evidence of publication bias (Egger’s *p* = 0.006). Ten studies including 152 participants reported the re-infection rate in patients who had undergone EF arthrodesis. There were 15 re-infections and the corresponding pooled re-infection rate was 7.2% (95% CI 2.3 to 12.1, *p* = 0.004). The heterogeneity between studies (I^2^ = 41%; *p* = 0.086) was lower in comparison with IM nail studies. When 1 single outlier was excluded [21], there was no more heterogeneity between studies and the pooled re-infection rate decreased to 5.4% (95% CI 1.7 to 9.1, p = 0.004) (Fig. 3). There was evidence of publication bias (Egger’s *p* = 0.001). The difference in re-infection rate between IM nail and EF once heterogeneity between studies was removed was significant in favour of EF arthrodesis (*p* = 0.009). When the effect of different surgical implants was analyzed using a subgroup analysis, the pooled re-infection rate of arthrodesis with short and long IM nail was 13.1% (95% CI 7.6 to 18.6) and 14.4% (95% CI 6.7 to 22.2), respectively, with no significant difference (Table 2). The heterogeneity of the model was moderate to substantial (I^2^ = 54%, *P* = 0.004). The subgroup analysis did not show any differences in re-infection rate between unilateral (7.5%; 95% CI 1.3 to 13.7) and biplanar/circular (8.5%; 95% CI 0.8 to 16.1) EF arthrodesis. The heterogeneity for this model was not significant (I^2^ = 27%, *p* = 0.179). The re-infection rate between EF with pins (5.4%; 95 CI 1.3 to 9.5) or wires (5.4%; 95% CI 3.1 to 14.0) was identical, once the heterogeneity between studies was eliminated (I^2^ = 0%; *p* = 0.990) by removing 1 outlier study [21] from the model. Details of the subgroups analysis for EF arthrodesis are provided in Table 3.

**Table 2:** Subgroup analysis of intramedullary nail arthrodesis studies

Outcome	Publications (*n*)	Cases	Long nail	Short nail	*P*
Re-infection	18	Total	77	345	
		No	65	291	0.998
		Yes	12	54	
Bone fusion	11	Total	77	112	
		No	13	14	0.397
		Yes	64	98	
Time to fusion (months ± SD)	8		5.62 ± 1.6	6.03 ± 0.6	0.626

*SD* Standard deviation

**Table 3:** Subgroup analysis of external fixator arthrodesis studies

Outcome	Publications (*n*)		Unilateral *n* = 66	Bipl/Circ *n* = 86	p	Pins *n* = 126	Wires *n* = 26	*P*
Re-infection	10	No	61	76	0.410	105^a^	25	0.745
		Yes	5	10		6^a^	1	
Bone fusion	10	No	11	13	0.795	23	1	0.066
		Yes	55	73		103	25	
Time to fusion (months ± SD)	10		7.60 ± 1.6	6.77 ± 0.5	0.314	7.38 ± 1.3	6.45 ± 0.2	0.086

*Bipl/Circ* Biplanar/Circular, *SD* Standard deviation

^a^1 outlier study removed

### Major complications and re-operations

Data for major complications (excluding re-infection) were pooled across 17 and 8 studies, respectively, for IM nail and EF arthrodesis. The most frequent major complication for IM nail and EF were implant failure and non-union, respectively. Pin track infection was reported with high frequency in all series of EFs but was not regarded as a major complication. The pooled random effects complication rate was 11.0% (95% CI 6.5 to 15.5, *p* < 0.001) for IM nail and 22.3% (95% CI 9.6 to 34.9, p < 0.001) for EF. Analysis of data revealed significant difference for treatment effect in favour of IM nail (p < 0.001). Heterogeneity between studies was substantial for both treatment strategies (IM nail – I^2^ = 66%; EF – I^2^ = 72%). Seventeen and 9 studies reported data regarding re-operations for arthrodesis with IM nail and EF, with a pooled random effects rate of 17.2% (95% CI 11.4 to 23.1, p < 0.001; I^2^ = 66%) and 19.3% (95% CI 9.4 to 29.3; p < 0.001; I^2^ = 66%), respectively. This difference was not significant (*p* = 0.447).

### Radiographic union

Overall, the rate of radiographic union was not significantly different between IM nail and EF arthrodesis but the mean time to fusion was shorter with IM nail (5.78 range 3.6–8.0 months vs. 7.19 range 6.3–10.3 months; *p* = 0.031). In detail, data on the radiographic union rate following arthrodesis with IM nail were obtained from 11 of 18 studies. No such data were available in more recent articles (i.e. after 2013). The pooled random effects union rate for IM nail arthrodesis was 89.4% (95% CI 84.1 to 94.8, *p* < 0.001), with non-relevant heterogeneity between studies (I^2^ = 40%; *P* = 0.082). The subgroup analysis did not show significant differences in rate of bone union following knee arthrodesis with short periarticular (91.2%; 95% CI 84.4 to 98.1) or long IM nail (86.1%; 95% CI 77.4 to 94.7) (Table 2). All 10 studies on EF investigated radiographic union as an outcome. The pooled random effects union rate for this surgical option was 87.9% (95% CI 81.0 to 94.9, *p* < 0.001), with moderate heterogeneity between studies (I^2^ = 57%; *p* = 0.013). On the exclusion of the two oldest studies,^21,25^ the pooled union rate for EF increased to 92.0% (95% CI 87.5 to 96.4, p < 0.001) and no heterogeneity was present (I^2^ = 0%; *p* = 0.484). Uniplanar and biplanar/circular EF had similar rate of bone fusion (Unilateral = 86.1%; 95% CI 77.1 to 95.1; biplanar/circular = 89.1%; 95% CI 81.4 to 96.8), but a nearly significant difference in this outcome was detected when EF with pins (85.8%; 95% CI 77.9 to 93.7) was compared to EF with wires (94.6 95% CI 86.0 to 103.1). The mean time to bone fusion also was shorter for the wire EF than for pin EF. Details of the subgroup analysis for EF arthrodesis are given in Table 3.

### Clinical outcomes

The comparison of clinical outcomes following IM nail and EF knee arthrodesis is reported in Table 4. With the numbers available, the only significant difference between these two surgical options was a bigger LLD in patients who had undergone arthrodesis with EF in comparison with those treated with IM nail. The extreme variability in assessment tools prevented us to perform any comparative analyses of knee functionality.

**Table 4:** Clinical Outcomes

		Intramedullary nail				External Fixator				*P* value
		Studies (*n*)	Patients (*n*)	Mean	SD	Studies (*n*)	Patients (*n*)	Mean	SD	
SF-36 or SF-12 questionnaire	PCS	3	94	35.0	8.7	2	38	41.1	2.5	0.357
	MCS	3	94	48.3	4.7	2	38	49.0	14.6	0.941
	PF	3	63	25.5	15.3	1	18	32.1		
	BP	3	63	43.8	17.8	1	18	47.4		
	RP	3	63	31.6	15.3	1	18	30.8		
	GH	3	63	45.9	10.3	1	18	46.9		
	SF	3	63	56.1	12.6	1	18	56.4		
	RE	3	63	45.4	24.7	1	18	46.7		
	VT	3	63	43.6	9.8	1	18	43.1		
	MH	3	63	63.5	11.8	1	18	58.3		
VAS pain		7	180	2.5	1.7	3	49	2.9	0.8	0.627
LLD (mm)		11	300	23.8	14.8	7	108	40.4	6.6	0.005

*SD* Standard deviation, *SF-36* Short form-36, *SF-12* Short form-12, *PCS* Physical component summary, *MCS* Mental component summary, *PF* Physical functioning, *BP* Bodily pain, *RP* Role-physical, *GH* General health, *SF* Social functioning, *RE* Role-emotional, *VT* Vitality, *MH* Mental health, *VAS* Visual analogue scale, *LLD* Limb length discrepancy

## Discussion

Knee arthrodesis with IM nail or EF is the most reliable therapeutic option to achieve definitive infection control in patients with septic failure of TKA [8, 9]. Deficient bone stock, impaired quality of bony surfaces, and shortened limbs may compromise the success of the procedure and lead to poor functional results [38]. To the best of our knowledge, this is the first review that compares IM nail and EF to achieve knee arthrodesis in the specific context of PJI. Indeed, two previous systematic reviews [11, 38] were not limited to studies of septic patients only. The most recent meta-analysis on the same topic [11] is different from our study for eligibility criteria and the primary endpoint. Indeed, White et al. [11] evaluated comparative studies that included also patients who had undergone knee arthrodesis for aseptic failure of TKA. The rate of radiographic union was assessed as a primary outcome. The inclusion criteria led these authors to select 12 comparative studies for the analysis, none of which was a randomized trial. Only two of these studies [10, 22] were used in our meta-analysis in that remaining studies did not fulfilled our stringent exclusion criteria because of methodological issues (e.g. mixed diagnosis and surgical strategy, small sample size, short follow-up period etc.). Conversely, we performed the meta-analysis by selecting studies that reported results of knee arthrodesis with EF or IM nail only in the specific context of septic failure of TKA. This approach has already be used in one previous meta-analysis that specifically dealt with the surgical revision of infected TKA [1]. Following previous studies [1], our primary outcome was the re-infection rate. Actually, infection control is the main goal of treatment when salvage surgery is performed for septic failure of TKA. Differently from the meta-analysis of White et al. [11], we assessed bone fusion as a secondary outcome. Indeed, achieving bone fusion may not be necessary in knee arthrodesis with the use of modular locking IM nails [33]. The eligibility criteria and selection of outcomes in the current analysis also enabled us to include studies on knee arthrodesis performed with the most recent modular IM nails that were completely excluded from the study of White et al. [11].

The present analysis showed that IM nail or EF arthrodesis have similar re-infection rates. However, most studies in the current meta-analysis only provided data on one of these two treatment strategies, which made internal matching of studied samples missing. This makes more important to remove outliers and reduce heterogeneity, so that fair comparisons between the two groups could be made. Actually, an excess rate of re-infection after IM nail arthrodesis became evident once outlier studies were dropped off from the meta-analysis. Increased risk of recurrent infection with IM nailing as compared to EF arthrodesis has been previously reported [9, 10], even though this finding is not supported by one recent meta-analysis [11]. Overall, these results suggest caution if the use of IM nailing is planned in difficult-to-treat PJIs (i.e. isolation of multi-resistant micro-organisms, multiple comorbidities etc).

The present meta-analysis has shown that patients who had undergone arthrodesis with IM nail have lower rate of major complications in comparison with those treated with EF, but the pooled rate of re-operation is similar. Besides major complications, the need for daily pin site care to prevent local complications represents a drawback when using the EF [9].

The rate of radiographic union in the studies included in this review was similar between IM nail and EF, differently from one recent meta-analysis [11] that found better results with IM nailing. With the numbers available, no differences in fusion rate emerged between long and short periarticular nails. Several recent studies reporting results of modular IM implants disregarded bony union as an endpoint since it was not considered essential to obtain successful outcome [5, 10, 12, 23, 26, 29, 33]. Conversely, bony union is of outmost importance for EF arthrodesis. Indeed, Corona et al. [18] showed that 82% of patients treated with EF who achieve fusion is satisfied with the result. Among those who do not achieve fusion, 75% is dissatisfied. No significant difference in the rate and time of bone fusion was detected when unilateral or biplanar/circular EF was used to obtain knee arthrodesis. However, there was a tendency toward better results using circular fixators and wires. Despite numerous disadvantages (frame maintenance, cosmetic discomfort, risk of neurovascular damage during wire insertion), circular external fixation offers possible progressive adjustment to stimulate the bony fusion while keeping maximum triplanar stability at the arthrodesis site [32]. The severe bone defect represents a specific problem following multiple revisions for PJI and direct bony union in these circumstances will result in marked LLD. The shortening of limb has detrimental effect on functional outcome [8], with a breakpoint of 2 to 3 cm [39]. Friedrich et al. [23] set a minus two centimeters to allow walking without circumduction of the leg after IM nailing. The mean LLD following IM nailing in the present analysis was about 2 cm and was significantly smaller than that recorded after EF arthrodesis, confirming previous positive results of modular nail without bone fusion [5]. No significant differences in the quality of life and severity of pain between the two surgical strategies were detected in this study, but these findings should be interpreted with caution in the context of the limited available data. Moreover, the paucity of literature data prevented us to perform a subgroup analysis that related the bone union to clinical outcomes. Previous studies reported moderate physical disability and mild mental disability after knee arthrodesis independent of the surgical strategy [17, 19]. Literature data for postoperative pain are inconclusive. Significant postoperative improvement in pain has been reported following IM nail arthrodesis [10, 16, 40], but other authors [12] obtained much worse results. Ramazzini-Castro et al. [41] found better result for pain in patients who had undergone arthrodesis with EF when compared to other surgical strategies.

The limitations of this study deserve attention. First, this meta-analysis was performed on cohort studies, because of the lack of randomized controlled trials on the outcome of knee arthrodesis with EF and IM nail. Hence, there was low quality of evidence for each outcome. Moreover, variable design and the different way to assess results may have contributed to the heterogeneity between studies that emerged for some outcomes assessed in the present meta-analysis. Nevertheless, once few outliers were excluded the heterogeneity disappeared. We also acknowledge the limited number of studies on EF arthrodesis. This limitation prevented us to perform a robust comparison between the two procedures, especially for secondary clinical endpoints, and to carry out a subgroup analysis to assess the influence of different factors (i.e. number of surgical stages, number of previous surgeries, bone fusion etc.) on the outcomes. Lastly, significant publication bias was identified in both treatment groups, which might undermine the conclusion of the study.

This study also shows different strengths. First, we adopted stringent eligibility criteria that led to the exclusion of studies that assessed results of knee arthrodesis for causes different from the septic failure of TKA. Actually, knee arthrodesis for PJI compels the surgeon to deal with specific clinical and microbiologic problems. Furthermore, unlike one recent meta-analysis [11], we included only studies with adequate sample size and follow-up interval. Indeed, studies with less than 10 participants are more likely to be case series which do not include consecutive patients [1]. Similarly, a follow-up time of less than 1 year is not suitable to compare decision-making outcomes in a meta-analysis [14]. Another strength of this study is the use of a validated instrument for non-randomized surgical studies to assess the methodological quality of studies included. Finally, we compared post-operative clinical outcomes that have not been considered in previous reviews [11, 38].

## Conclusions

The available evidence suggests that knee arthrodesis with EF in the specific context of PJI has a reduced risk of re-infection in comparison with the IM nail strategy. Hence, caution should be exercised particularly when the use of IM nail is planned in difficult-to-treat PJIs. The use of IM nail is more advantageous than EF with respect to important clinical outcomes such as the frequency of major complications and postoperative LLD.

## Abbreviations

CI: Confidence interval
EF: External fixator
IM: Intramedullary
LLD: Limb length discrepancy
MINORS: Methodological Index for Non-Randomized Studies
PJI: Periprosthetic joint infection
PRISMA: Preferred Reporting Items for Systematic Review and Meta-Analyses
SF-12: 12-item short form health survey
SF-36: 36-item short form health survey
TKA: Total knee arthroplasty
